# Data for improvement and clinical excellence: report of an interrupted time series trial of feedback in long-term care

**DOI:** 10.1186/s13012-014-0161-5

**Published:** 2014-11-11

**Authors:** Anne E Sales, Corinne Schalm, Melba Andrea B Baylon, Kimberly D Fraser

**Affiliations:** Center for Clinical Management Research, VA Ann Arbor Healthcare System, Ann Arbor, MI USA; Division of Nursing Business and Health Systems, School of Nursing, University of Michigan, 400 N. Ingalls Street, Ann Arbor, MI 48109-5482 USA; Alberta Health and Wellness, Edmonton, Alberta Canada; Faculty of Nursing, University of Alberta, Edmonton, Alberta Canada

**Keywords:** Audit with feedback intervention, Interrupted time series, Long-term care, Quality improvement

## Abstract

**Background:**

There is considerable evidence about the effectiveness of audit coupled with feedback for provider behavior change, although few feedback interventions have been conducted in long-term care settings. The primary purpose of the Data for Improvement and Clinical Excellence-Long-Term Care (DICE-LTC) project was to assess the effects of a feedback intervention delivered to all direct care providers on resident outcomes. Our objective in this report is to assess the effect of feedback reporting on rates of pain assessment, depression screening, and falls over time.

**Methods:**

The intervention consisted of monthly feedback reports delivered to all direct care providers, facility and unit administrators, and support staff, delivered over 13 months in nine LTC units across four facilities. Data for feedback reports came from the Resident Assessment Instrument Minimum Data Set (RAI) version 2.0, a standardized instrument mandated in LTC facilities throughout Alberta. The primary evaluation used an interrupted time series design with a comparison group (units not included in the feedback intervention) and a comparison condition (pressure ulcers). We used segmented regression analysis to assess the effect of the feedback intervention.

**Results:**

The primary outcome of the study, falls, showed little change over the period of the intervention, except for a small increase in the rate of falls during the intervention period. The only outcome that improved during the intervention period was the proportion of residents with high pain scores, which decreased at the beginning of the intervention. The proportion of residents with high depression scores appeared to worsen during the intervention.

**Conclusions:**

Maintaining all nine units in the study for its 13-month duration was a positive outcome. The feedback reports, without any other intervention included, did not achieve the desired reduction in proportion of falls and elevated depression scores. The survey on intention to change pain assessment practice which was conducted shortly after most of the feedback distribution cycles may have acted as a co-intervention supporting a reduction in pain scores. The processing and delivery of feedback reports could be accomplished at relatively low cost because the data are mandated and could be added to other intervention approaches to support implementation of evidence-based practices.

**Electronic supplementary material:**

The online version of this article (doi:10.1186/s13012-014-0161-5) contains supplementary material, which is available to authorized users.

## Background

Long-term care (LTC) settings, also described as nursing homes or long-term care facilities, exist in some form in most developed countries. In Canada and the United States, institutional long-term care facilities are usually stand-alone facilities or sometimes part of a hospital. They provide ongoing, continuing care to physically frail, often cognitively impaired, residents who live there, for the most part, until their death. Many facilities provide both rehabilitation and long-term care [1]. The focus of our project was on LTC units, not those providing post-acute care or other rehabilitation services. LTC units usually operate under resource constraints and rely heavily on non-professional staff to provide the majority of care to physically frail and typically cognitively impaired older adults. In these settings, resources for quality improvement are difficult to obtain, and trials of quality improvement interventions are relatively rare compared with other health-care settings.

There is a growing evidence base for care in LTC settings, including evidence-based approaches to pain management, falls prevention, and other important topics [2-5]. However, getting evidence into practice is not well understood, and relatively little evidence exists to date on effective interventions for implementing evidence-based practices and changing provider behavior in these settings [6]. Many interventions have been rigorously tested across multiple settings and conditions, and some evidence exists for the effectiveness of specific interventions in implementing evidence-based practice through changing provider behavior [7-10]. One of these is audit combined with feedback reports (referred to as feedback interventions). Feedback interventions have demonstrated modest effect in promoting desired behavior change among health-care providers across settings and provider types [11,12]. To date, few feedback interventions have been conducted in LTC settings. Those that have been conducted have included only a subset of the providers who give care to residents, typically the most educated professionals [13]. A major barrier to using feedback interventions in LTC has been the lack of data on care processes and outcomes, but this has been addressed in many jurisdictions by the adoption of a Minimum Data Set which assesses resident care processes and outcomes [14-17].

The probable mechanism by which feedback interventions have their main effect is in providing people with information about their own performance [18-20]. The results, particularly among people who have not received data-based feedback on their performance in the past, may be to increase motivation to change behavior. Feedback reports have often been used in conjunction with other interventions [11]. Our intention in conducting this study was to keep the intervention simple and relatively low cost by using feedback reports alone without other interventions. The low cost of the type of feedback intervention we studied is supported by widespread availability of data on resident status and outcomes [14,15], which do not require significant additional cost for audit data to construct the feedback reports [14,15,21]. In this paper, we report the summative resident outcomes of a feedback intervention delivered to all staff in four LTC facilities. Our primary hypothesis was that a consistent, long-duration, resident-focused feedback intervention would improve resident outcomes compared with settings which did not receive the feedback intervention and compared with an outcome not included in the feedback report.

## Methods/Design

We used an interrupted time series design as the overall intervention evaluation approach with comparisons to assess the effect of monthly feedback reports in nine LTC units in four facilities. We conducted a concurrent process evaluation to evaluate staff response to the intervention. We report these results in a separate paper, currently under review. The project received ethics approval from the Health Research Ethics Board, Committee B, at the University of Alberta and operational approval from the two LTC organizations participating in the study. The full protocol for the entire Data for Clinical Improvement and Excellence (DICE) project was published in two protocol papers [22]. We provide brief descriptions of key elements in this paper, in which we report on the DICE-LTC (Long-Term Care) component of the full project [22].

As we describe in our protocol paper for a social network sub-study within DICE-LTC [22], we worked from an underlying conceptual model built on the Theory of Planned Behavior, in which we posited that feedback reports would work through multiple paths (attitudes and social norms in particular) to influence intention to change behavior. We were unable to measure provider behavior directly through this study, but we measured longitudinal measures of intention to change behavior, reported in the process evaluation paper. We expected an effect at the level of the LTC nursing unit, a geographic sub-unit of the entire facility, which is relatively self-contained with respect to most of the frontline, day-to-day, and shift-to-shift direct care providers, most of whom are health-care aides (HCAs). The geographic boundaries of the nursing unit are quite permeable, and other types of providers, notably those in the allied health professions including occupational, physical, and recreational therapists, social workers, and others, traverse unit boundaries. However, in our study, geographic nursing units all had a single, clearly identified unit manager and were understood as important and relatively independent components of the organization by staff in all four facilities. Our intervention included handing feedback reports to individual providers of all kinds, as there were few opportunities to deliver reports to groups of staff. We did, however, expect that providers would talk to each other about the reports, and we asked them questions to elicit an understanding of how much they discussed the reports, as well as some information about the purposes of the discussion, described in the process evaluation paper.

### Settings and sample

The project was conducted in nine LTC nursing units in four continuing care facilities in Edmonton, Alberta, Canada. The four facilities were part of two organizations providing a range of continuing care services in the community. All four facilities had implemented the Resident Assessment Instrument Minimum Data Set (RAI) version 2.0 (http://www.interrai.org), although two facilities had implemented RAI 2.0 between 3 and 5 years prior to the beginning of the DICE-LTC feedback intervention, while the other two had only implemented this system between 6 and 12 months prior to the project beginning. We initially hypothesized that maturity of RAI assessments might be an important factor in the uptake and effectiveness of the feedback reports.

### The intervention

The feedback reports were developed during a pilot study conducted in two nursing homes in the Edmonton area in late 2007 and early 2008. We used data from the RAI 2.0 to measure resident-level outcomes, and this served as the data source for the feedback reports. The RAI 2.0 assessment tool covers a wide range of process and outcome data at the individual resident level, and assessments are updated quarterly for each resident. We reported on measures of pain frequency and intensity, risk for and occurrence of falls, and depression prevalence, all aggregated to the unit level. These three areas were among the top eight domains identified as priorities through the pilot project and were agreed upon by senior leadership in both participating organizations. Data were extracted at the resident level from vendor servers every month and stripped of personal identifiers, except for the unit on which each resident lived, before being sent to the research team.

Following definitions used by the Canadian Institute for Health Information, we derived the pain scale from two items on the RAI 2.0 assessment, one measuring pain frequency and the other measuring pain intensity. The scale is scored 0 for no pain, 1 for pain less than daily, 2 for daily pain of moderate intensity, and 3 for daily severe pain [23]. The depression rating scale is scored on a 0 to 14 range and is derived from seven RAI 2.0 items, from making negative statements to crying and tearfulness. A score of 3 or more indicates possible depressive disorder and residents should be further evaluated [24]. Falls were defined as any fall occurring in the 31 to 180 days prior to the current assessment, and falls risk were defined as a combination of requiring assistance for locomotion, problems with balance, dizziness or vertigo, and unsteady gait. The assessments were completed by staff in each facility following comprehensive instructions and training required for RAI 2.0 assessments. We aggregated these into proportions for residents on each of the nine units.

Reports were primarily graphic on one sheet of paper, front and back, printed in color, and included a cover sheet with details about the data sources and the comparison units. We provided a sample of this report in the DICE-LTC protocol paper [22]. We provided feedback using monthly time points from months 2 to 11, after which we switched to showing quarterly time points for months 12 and 13. Reports were hand-delivered by project staff, who were all research assistants with minimal training, in each of the nine LTC units during a consistent week in each month for each of the 13 months of the intervention period. Each report was specific to the unit, and all direct care providers and unit managers received the unit-specific reports. Facility administrators received reports for each of their units prior to report distribution on the units. We included facility administrators, nurse managers, and frontline direct care staff, including registered nurses, licensed practical nurses, HCAs, physical therapists, recreational therapists, occupational therapists, pharmacists, social workers, and other allied health providers. The goal of the feedback report distribution was to ensure that frontline staff received the reports directly.

In addition to the feedback intervention, research assistants also distributed and collected post-feedback surveys in each intervention unit as part of an extensive process evaluation. In the first section of the post-feedback survey, we asked questions about response to the reports. In another section, we asked about intentions to change behavior with respect to assessing pain among residents. A sample survey instrument is provided in the protocol paper [22], and the results detailing provider response to the feedback reports are reported in the process evaluation paper.

### Comparison data

In addition to the data included in the feedback reports delivered to participating units and facilities, we also requested data from the same period for nine units in three additional facilities, matched as closely as possible to the facilities participating in the study. All three comparison facilities came from one of the two larger organizations participating in the study. These provided comparison units to control for secular trend over the baseline, intervention, and follow-up periods. We also included pressure ulcer prevalence, a quality indicator not included on the feedback reports, as an additional check on secular trends within the participating units.

### Analysis

Our primary analysis used segmented regression on the interrupted time series data. In this approach, all data are aggregated to a single observation at multiple time points at equal intervals. In our case, they were in months [25]. Given a data set aggregated to equal interval time points, we specify our linear regression model as:

$$ \begin{array}{l}{Y}_t={b}_0+{b}_1*{\mathrm{time}}_t+{b}_2*{\left(\mathrm{feedback}\right)}_t+{b}_3*{\left(\mathrm{time}\ \mathrm{after}\ \mathrm{intervention}\ \mathrm{starts}\right)}_t+{b}_4\kern0.5em *\\ {}{\left(\mathrm{end}\ \mathrm{of}\ \mathrm{intervention}\right)}_t+{b}_5*{\left(\mathrm{time}\ \mathrm{after}\ \mathrm{intervention}\ \mathrm{ends}\right)}_t+{e}_t\end{array} $$

where

- *Y*_*t*_ is the outcome in month *t*;
- *time* is the time in months at time *t* from the start of the observation period; it ranges from 1 to 25 months;
- *feedback* is an indicator for time *t* occurring before (feedback = 0) or after (feedback = 1) the feedback report, which was implemented in January 2009 in the series;
- *time after intervention starts* is the number of months after the intervention starts at time *t*, coded 0 before the feedback intervention starts and (time-6) after the feedback intervention starts;
- *end of intervention* is an indicator for time *t* occurring before or after the end of the feedback intervention, which was after January 2010 in the time series;
- *time after intervention ends* is the number of months after the intervention at time *t*, coded 0 before the end of the feedback report and (time-19) after the end of the feedback report;
- e_*t*_ is the error term at time *t* that represents the unexplained random variation in the model.

In this model, we estimated the regression parameters as follows:

- *b*_0_ is the baseline level of the outcome;
- *b*_1_ is the slope of the regression line (trend line) prior to the feedback report;
- *b*_2_ is the change in level immediately following the feedback report start;
- *b*_3_ is the change in regression slope during the feedback report period;
- *b*_4_ is the change in level immediately after the feedback report ends;
- *b*_5_ is the change in regression slope in the post-feedback report period.

We conducted separate segmented regression analyses for the intervention and comparison facilities in each of the four outcomes: proportion of residents with pain scores greater than 2, proportion with depression scores greater than 3, proportion with falls, and proportion with pressure ulcers. We provide both visual representations of the results in the form of time series graphs and tables of the parameter estimates from the regression analyses for statistical inference. We tested the residuals from each regression analysis for autocorrelation using the Durbin-Watson test, and if this was significant, we used the Prais-Winsten regression to adjust for autocorrelation [26,27]. The Durbin-Watson test is a check of first-order serial auto-correlation due to repeated measures (AR1); the Prais-Winsten regression adjusts for first-order serial auto-correlation by assuming an AR1 error term. When the Durbin-Watson test did not show significant autocorrelation, we present the results of ordinary least squares regression of the time series data. Ordinary least squares regression is more commonly termed linear regression. In each month, we included only residents who had received a new assessment during that month, which lessened the degree of autocorrelation between months as different resident data were included each month. We used this approach in the monthly feedback reports also, to increase the degree to which the data would change from month to month and provide new information.

We initially powered our sample size on falls as the primary outcome as we designed the project, using data reported in the literature on the prevalence of falls in LTC settings similar to those included in our study, and on effect sizes for change in falls due to quality improvement interventions similar to our planned feedback intervention. We did not, in our original power calculations, focus on the interrupted time series design, but instead used a standard approach with an adjustment for clustering. However, our final sample included all available LTC units in the four facilities participating in the project. While the number of residents across all available units met our calculated sample size in designing the project, we did not take into account the interrupted time series approach to analysis. As a result, our sample size calculation may have overestimated our power. In addition to the primary analyses, we also conducted secondary analyses dividing the intervention units into those with mature RAI 2.0 systems compared to those with recent implementation of RAI 2.0. We included secondary hypotheses about the effects of mature vs. new data systems in our original proposal [22].

**Figure 1:**
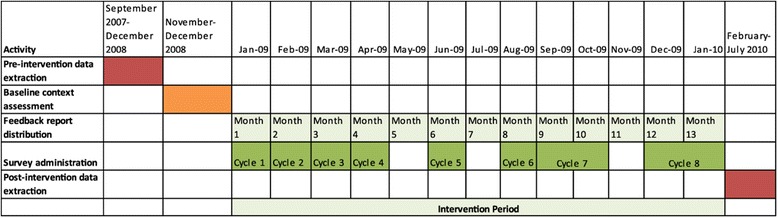
**Timeline for DICE-LTC.**

We did not risk-adjust our outcomes. We were focused on reporting observed data to the staff providing care to residents, and while risk adjustment might be necessary for comparing across units for performance management or reporting purposes, for quality improvement, staff needed to understand the experience of residents in their care. The data used in this summative evaluation are the same as those provided to staff throughout the feedback intervention. We included data from 6 months prior to the start of the feedback intervention, which began in January 2009, as pre-intervention data, and from 6 months after the end of the intervention, as post-intervention data. The timeline in Figure 1 shows the timing of data extraction, feedback report delivery, and post-intervention data.

### Variables included in the analysis

Following the segmented regression analysis approach, the only variables included in these analyses are the time points based on the study timeline and outcome data at each time point. The intervention and comparison units are implicit in the different regression analyses.

## Results

### Resident characteristics

Over the full 13 months of the intervention, we reported data on over 500 unique residents in nine LTC nursing units in four facilities participating in the feedback intervention. In Table 1, we present data describing the resident sample from the intervention units at four relevant time periods: at the beginning of the pre-intervention period (July 2008), at the beginning of the intervention period (January 2009), at the end of the intervention period (January 2010), and at the end of the post-intervention period (July 2010). As generally observed among LTC residents, the majority were female and most were widowed. In Table 2, we provide similar data for residents from the comparison units. Numbers are similar in each period, as is the proportion of female residents. In Tables 3 and 4, we show the number of residents included in the monthly feedback reports by nursing unit and facility at important time points in both the intervention and comparison sites.

**Table 1:** **Overall intervention site resident demographics at four time points: pre-intervention, beginning of intervention period, end of intervention period, and post-intervention**

**Demographic characteristics**	**July 2008**		**January 2009**		**January 2010**		**July 2010**	
	**(** ***n*** **= 111)**		**(** ***n*** **= 139)**		**(** ***n*** **= 157)**		**(** ***n*** **= 145)**	
Age (in years)								
Mean	84.7		84.2		85.7		86.3	
Standard deviation	7.9		8.7		8.6		7.6	
Range	48 to 102		43 to 103		45 to 104		60 to 102	
Sex	No.	%	No.	%	No.	%	No.	%
Male	38	34.2	42	30.2	45	28.7	40	27.6
Female	73	65.8	97	69.8	112	71.3	105	72.4
Marital status	No.	%	No.	%	No.	%	No.	%
Never married	4	3.6	6	4.3	5	3.2	7	4.8
Married	28	25.2	39	28.1	42	26.8	32	22.1
Widowed	70	63.1	82	59.0	99	63.1	94	64.8
Separated	1	0.9	1	0.7	─	─	1	0.7
Divorced	8	7.2	11	7.9	11	7	10	6.9
Unknown	─	─	─	─	─	─	1	0.7

**Table 2:** **Overall comparison site resident demographics at four time points: pre-intervention, beginning of intervention period, end of intervention period, and post-intervention**

**Demographic characteristics**	**July 2008**		**January 2009**		**January 2010**		**July 2010**	
	**(** ***n*** **= 132)**		**(** ***n*** **= 122)**		**(** ***n*** **= 114)**		**(** ***n*** **= 145)**	
Age (in years)								
Mean	79.5		79.4		78.7		80.8	
Standard deviation	13.6		13.7		14.6		13.5	
Range	49 to 102		49 to 102		38 to 100		42 to 101	
Sex	No.	%	No.	%	No.	%	No.	%
Male	38	28.8	41	33.6	38	33.3	36	30.8
Female	94	71.2	81	66.4	76	66.7	81	69.2
Marital status	No.	%	No.	%	No.	%	No.	%
Never married	17	12.9	14	11.5	16	14.0	13	11.1
Married	36	27.3	32	26.2	31	27.2	28	23.9
Widowed	61	46.2	55	45.1	52	45.6	61	52.1
Separated	2	1.5	2	1.6	1	0.9	1	0.9
Divorced	12	9.1	14	11.5	9	7.9	12	10.3
Unknown	4	3.0	─	─	5	4.4	2	1.7

**Table 3:** **Intervention site number of residents included in the reports at key time points by unit and facility (U** ***x*** **= unit designator; F** ***x*** **= facility designator; there were a total of nine units in four facilities within two organizations)**

	**July 2008**	**January 2009**	**January 2010**	**July 2010**
Unit				
U1F1	15	13	18	15
U2F1	16	16	12	10
U3F1	21	16	17	17
U1F2	12	10	10	9
U2F2	11	15	19	17
U3F2	12	12	14	14
U1F3	11	22	24	20
U2F3	7	24	24	26
U1F4	6	11	19	17
Total	111	139	157	145
Organization				
Org1 (facility 1 + facility 2)	87	82	90	82
Org2 (facility 3 + facility 4)	24	57	67	63
Total	111	139	157	145

**Table 4:** **Comparison site resident numbers included in the reports at key time points by unit and facility (CU** ***x*** **= control unit facility identifier; CF** ***x*** **= control facility identifier)**

	**July 2008**	**January 2009**	**January 2010**	**July 2010**
Unit				
CU1F1	10	8	7	6
CU2F1	7	5	7	7
CU3F1	8	7	10	6
CU4F1	12	11	13	11
CU1F2	10	11	9	12
CU2F2	28	25	26	27
CU3F2	25	27	23	28
CU1F3	17	14	9	10
CU2F3	15	14	10	10
Total	132	122	114	117
Facility				
CF1	37	31	37	30
CF2	63	63	58	67
CF3	32	28	19	20
Total	132	122	114	117

### Segmented regression analysis

**Figure 2:**
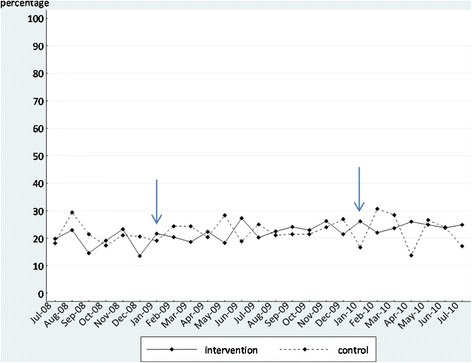
**Proportion of residents in the intervention and control groups who experienced a fall (in the past 31 to 180 days) before, during, and after the feedback intervention.**

**Figure 3:**
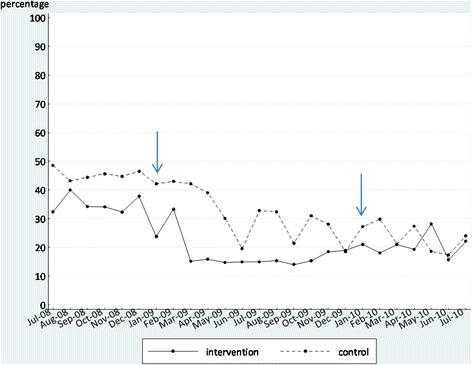
**Proportion of residents in the intervention and control groups with moderate to severe pain before, during, and after the feedback intervention.**

**Figure 4:**
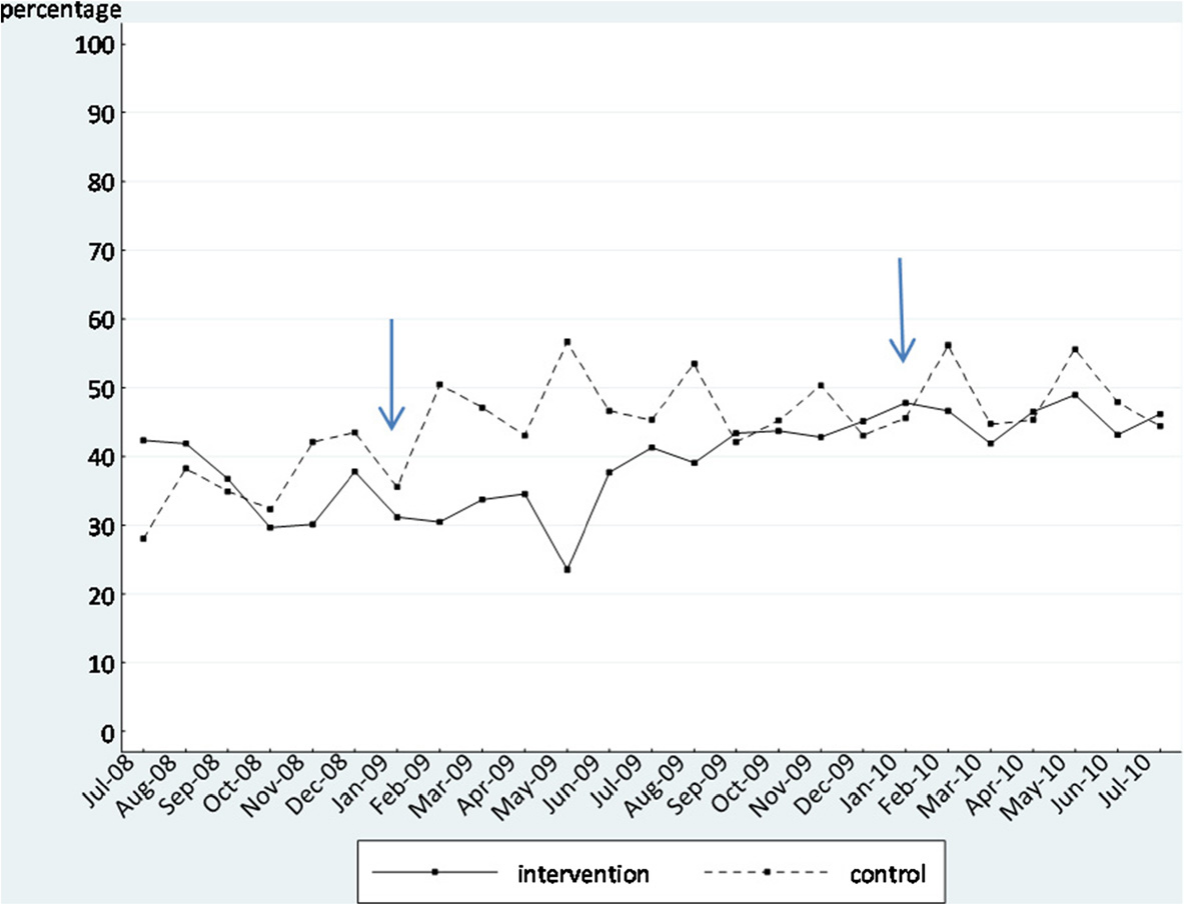
**Proportion of residents in the intervention and control groups with depression who needed further evaluation before, during, and after the feedback intervention.**

**Figure 5:**
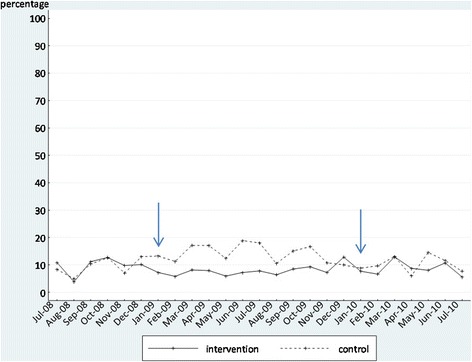
**Proportion of residents in the intervention and control groups who had pressure ulcers before, during, and after the feedback intervention.**

We initially display the time series as graphs in Figures 2, 3, 4, and 5. These graphs allow the reader to evaluate the absolute levels of the outcomes in each instance, as well as the change over time. In each graph, the blue arrows indicate the beginning and end of the intervention period. In Tables 5 and 6, we summarize the findings from the segmented regression analyses for the intervention and comparison sites, respectively. The Durbin-Watson statistic was significant only for one outcome, falls, in the intervention sites. As a result, we present the Prais-Winsten regression results for falls. The other regression results are all from ordinary least squares regression.

**Table 5:** **Segmented regression results for intervention sites**

	**Coefficient estimate**	***t*** **-statistic**	***p*** **-value**	**Lower limit 95% confidence interval**	**Upper limit 95% confidence interval**
Falls					
*b*_0_	20.89	13.70	<0.01	17.70	24.08
*b*_1_	−0.50	−1.28	0.22	−1.33	0.32
*b*_2_	1.46	0.98	0.34	−1.67	4.58
*b*_3_	0.93	2.31	0.03	0.09	1.77
*b*_4_	−1.41	−0.81	0.43	−5.05	2.22
*b*_5_	−0.16	−0.39	0.70	−1.00	0.68
Pain score					
*b*_0_	34.76	7.62	<0.01	25.21	44.31
*b*_1_	0.11	0.10	0.92	−2.34	2.57
*b*_2_	−14.32	−3.13	0.01	−23.89	−4.75
*b*_3_	−0.54	−0.44	0.66	−3.11	2.03
*b*_4_	3.80	0.73	0.48	−7.16	14.76
*b*_5_	0.81	0.66	0.52	−1.76	3.38
Depression score					
*b*_0_	42.93	11.82	<0.01	35.33	50.53
*b*_1_	−1.86	−1.99	0.06	−3.81	0.09
*b*_2_	−4.44	−1.22	0.24	−12.06	3.17
*b*_3_	3.39	3.47	0.00	1.34	5.43
*b*_4_	−1.99	−0.48	0.64	−10.71	6.74
*b*_5_	−1.42	−1.45	0.16	−3.46	0.63
Pressure ulcers					
*b*_0_	5.47	3.02	<0.01	1.67	9.26
*b*_1_	0.05	0.11	0.92	−0.92	1.02
*b*_2_	−3.41	−1.88	0.08	−7.21	0.39
*b*_3_	0.28	0.58	0.57	−0.74	1.30
*b*_4_	0.48	0.23	0.82	−3.87	4.83
*b*_5_	−0.60	−1.24	0.23	−1.62	0.42

**Table 6:** **Segmented regression results for comparison sites**

	**Coefficient estimate**	***t*** **-statistic**	***p*** **-value**	**Lower limit 95% confidence interval**	**Upper limit 95% confidence interval**
Falls					
*b*_0_	23.04	5.53	<0.01	14.31	31.76
*b*_1_	−0.49	−0.45	0.65	−2.73	1.75
*b*_2_	2.71	0.65	0.52	−6.03	11.45
*b*_3_	0.43	0.38	0.71	−1.92	2.77
*b*_4_	8.20	1.71	0.10	−1.81	18.21
*b*_5_	−1.90	−1.70	0.11	−4.25	0.44
Pain score					
*b*_0_	45.86	9.78	<0.01	36.05	55.68
*b*_1_	−0.10	−0.08	0.93	−2.62	2.42
*b*_2_	−2.49	−0.53	0.60	−12.32	7.35
*b*_3_	−1.53	−1.21	0.24	−4.17	1.11
*b*_4_	6.45	1.20	0.25	−4.82	17.71
*b*_5_	0.22	0.17	0.87	−2.42	2.86
Depression score					
*b*_0_	27.88	5.59	<0.01	17.44	38.31
*b*_1_	2.47	1.93	0.07	−0.21	5.15
*b*_2_	3.04	0.61	0.55	−7.42	13.49
*b*_3_	−2.35	−1.76	0.10	−5.16	0.45
*b*_4_	5.68	0.99	0.33	−6.30	17.66
*b*_5_	−1.21	−0.91	0.38	−4.02	1.59
Pressure ulcers					
*b*_0_	3.53	1.91	0.07	−0.35	7.41
*b*_1_	0.14	0.30	0.77	−0.86	1.14
*b*_2_	1.50	0.81	0.43	−2.38	5.39
*b*_3_	−0.21	−0.41	0.68	−1.25	0.84
*b*_4_	−1.41	−0.67	0.51	−5.87	3.04
*b*_5_	0.02	0.04	0.97	−1.02	1.07

#### Falls

The coefficient estimate for the change in regression slope during the intervention period (*b*_3_) in the intervention sites is significant but positive, indicating that the rate of falls increased more during the intervention than it had prior to the intervention, counter to our expectation that falls would decrease during the intervention period. None of the other coefficient estimates for falls were significant.

#### Pain

For the proportion of residents with high pain scores, by contrast, the coefficient estimate for the change in level when the intervention started (*b*_2_) is significant and negative, indicating that there was a decrease in the level of the pain scores at the beginning of the intervention. Though the change in regression slope coefficient estimate during the intervention phase (*b*_3_) was negative, it was not statistically significant, indicating that the slope during the intervention period was not significantly different from the slope prior to the intervention, and none of the other parameter estimates were significant.

#### Depression

For the proportion of residents with high depression scores, the pre- and post-intervention regression slope coefficient estimates (*b*_1_ and *b*_5_, respectively) were negative and insignificant but the change in slope during the intervention period (*b*_3_) was positive and significant, as for falls. Again, this was counter to our expectation.

#### Pressure ulcers

For the proportion of residents with pressure ulcers, which was a control condition not included in our feedback reports, no parameter estimates were significant. We expected no change in pressure ulcer levels as they were not included in our feedback reports.

#### Comparison sites

In the comparison sites, there were no significant changes, either positive or negative, in any of the coefficient estimates for any of the indicators. This is what we expected, because we did not deliver feedback reports in any of these LTC units.

In our secondary analyses of units with more mature vs. more recent implementation of RAI 2.0 (provided as Additional files 1 and 2), we saw a mixed pattern of results across the different outcomes, with little statistical significance. As a result, it does not appear that maturity of data systems had a consistent effect on outcomes.

## Discussion

We report on a feedback intervention which we were able to deliver consistently over a 13-month period across nine LTC units in four facilities. Given the low level of resources available in these facilities, completing the intervention with all nine original units, and with a relatively high rate of participation among providers, is an important achievement. Attrition rates for quality improvement interventions is a critical problem among LTC facilities, usually attributed to high rates of staff turnover, low levels of education, and high workload, and many quality improvement interventions have not been able to retain all participating units for the full project [6].

Based on our primary outcome, falls, the feedback intervention was not effective in changing resident outcomes. We had hoped to demonstrate a reduction in falls as a result of the intervention. Instead, we found no immediate change in the level or number of falls at the outset of the intervention, when the effect of feedback might be highest, and a modest but significant increase in the rate of falls over the intervention period. This latter finding was contrary to our expectation. The lack of any significant change in either the control condition (pressure ulcers) or comparison sites suggests that there were few, if any, secular trends during the intervention period which would have changed resident outcomes.

In strict terms, then, we did not find the positive effect of feedback interventions alone that we had hoped to find. This is consistent with the literature on feedback interventions, which suggests that feedback interventions are likely insufficient by themselves to effect sufficient provider behavior change to change outcomes. This is mediated significantly by the degree of uptake of the feedback report, the conditions under which it was received, and other factors, based on the Theory of Planned Behavior, which might result in behavior change following receipt of a feedback intervention. In addition to factors related to the Theory of Planned Behavior, which include attitudes and beliefs, social and professional norms, and perceived behavioral control [28], factors related to the feedback intervention itself are likely important. These include the timeliness of the data, the perceived validity of the data, the source of the feedback, and other factors such as the perceived sign of the information in the report (feedback perceived as positive, negative, or neutral) [22]. We describe findings related to a number of these factors in the process evaluation paper. Briefly, a high proportion of the target provider groups received, read, and understood the feedback report. A smaller proportion discussed it with other staff in the unit. Many participants described an interest in receiving further data as well as further information about how to make changes to benefit residents, but it was beyond the scope of this project to provide information. The lack of time and opportunity to spend time in activities other than offering direct care to residents was a serious and continual constraint on our activities.

Despite that, we note that there was a significant change in pain scores at the beginning of the intervention period, in the direction we had expected. It is notable that the post-feedback survey we administered 1 week after the feedback report distribution asked a set of questions about intention to change pain assessment behavior among direct care staff. We report on these findings in the companion process evaluation paper, but note that this may have unintentionally worked as a co-intervention with the feedback report, as it may have acted to focus participant attention on pain assessment. While this was an unexpected effect, not one we intentionally designed into the intervention, similar results have been observed previously, and there is a literature on what is called “mere measurement” or the “question-behavior effect” [29,30]. This is an intriguing finding that deserves follow-up work in future implementation studies.

We focused on pain assessment because it had been ranked highly as an important quality improvement issue across all provider groups in our pilot study and had been the subject of attention in both organizations participating in the intervention. Managers strongly endorsed pain assessment as an important issue for measurement, and prior to initiating the feedback intervention, several sites had engaged in education about pain assessment and management. The graphical time series for this outcome depicted in Figure 3 shows that the intervention sites demonstrated a decrease in proportion of residents with high pain scores at the outset of the intervention, followed by a period of several months of low scores which slowly climbed again towards the end of the intervention period. In the comparison sites, there was a later drop in scores, but then an inconsistent pattern of increasing and decreasing scores over most of the intervention period. From the coefficient estimates, we see that the change in scores at the beginning of the intervention period was significant for the intervention units, while the changes for the comparison units were not, even though the comparison units were given the same education, unrelated to our intervention, prior to the intervention period.

An important note is that this is the first attempt, to our knowledge, to deliver feedback reports to all direct care providers in LTC settings, including health-care aides. Most previous studies have excluded aides, often without justification. The justifications that have been used frequently include feasibility, lack of educational preparation, and that the primary drivers of quality improvement are professional providers, usually registered or licensed practical nurses. We argue that because aides are the majority of providers in LTC settings, excluding them from involvement in quality improvement interventions may have unintended negative consequences and may decrease the effectiveness of the intervention. In this report, and in the companion process evaluation paper, we have demonstrated that including aides is feasible and that aides report being able to understand feedback reports.

### Limitations

Although this was one of the longest and most intensive studies of a feedback intervention in LTC conducted to date, with a strong process evaluation concurrent with a rigorous quasi-experimental design, our inferences are limited by a number of important issues. First, we were unable to obtain data to link intention to change to actual behavior change. We decided not to request identifying data on individual providers responding to surveys to avoid raising concerns about confidentiality and coercion, and we did not have data that would have allowed us to observe actual practice change leading to resident outcomes. Examples of these kinds of data include drug prescription and administration data for pain management, interventions undertaken to reduce resident risk of falls, prescribing and administering anti-depressant medication, or increases in activities to reduce resident isolation. Alternative approaches to data collection might have included more intensive observation of resident care, timed to coincide with report distribution. Prescription data, and data about direct interventions for residents, are not part of the RAI 2.0 assessment system in the facilities in which we conducted the project, making it impossible to obtain these data without considerable cost and identification of residents. We discussed more intensive observation of resident care with the decision makers on our research team, and the possible invasion of resident privacy, as well as the burdens placed on busy, often crowded, and sometimes stressful environments, precluded our ability to conduct more observations.

Another important limitation was the relatively small time series. Ideally, an interrupted time series design would include at least 40–50 time points measuring the dependent variables. We were able to include only 25 time points, which resulted in less than optimal power. The limitations were the lack of data prior to the start of the intervention. In two of the organizations in which we conducted the intervention, the RAI assessments only began shortly before the intervention started. We collected as much data as possible prior to the intervention. After the intervention ended, budget cuts reduced the number of staff available to support data extraction in both participating organizations, making it very difficult to obtain follow-up data. Difficulties of this kind are common in LTC, making it difficult to find resources to conduct intensive quality improvement interventions. As a result of the realities of data access in this study, we had a time series with 6 time points before the intervention started, 13 during the intervention, and 6 after the intervention ended. Ideally, we would have had a minimum of 10 time points during each phase. In addition, the number of residents included in the measures at each time point varied quite widely from 111 to 157, which created considerable variability, and may have affected the stability of the time series and the level of uncertainty around each time point.

## Conclusion

Most prior quality improvement efforts in LTC settings have not included control or comparison groups, and most have used pre- and post-intervention designs that make causal attribution very difficult. We included two different kinds of comparisons to guard against both attention effects and secular trend. We ruled out secular trend as a cause of the significant changes we saw. We also saw relatively little change in the desired direction as a result of this modest, intentionally parsimonious intervention. The one change in the desired direction we did find may have been related to an unintended co-intervention, namely the focus on pain assessment in our measurement instruments. It is difficult, based on our findings, to argue for continuing to conduct feedback interventions alone in LTC settings, although feedback reports can be an important building block in intervention design. Especially when data already exist, this can be a relatively low-cost component to an intervention that may enhance the effect of additional components such as education, goal setting, and other relevant behavior change techniques [31,32].

We believe that our findings should provide qualified evidence for managers on the usefulness of existing data from sources like the RAI 2.0, which are increasingly available in many jurisdictions. A major impetus for our project was the desire to use the data that staff so arduously collect on a quarterly basis, to improve the quality of care and quality of life of residents. We have shown that this work is feasible, although achieving significant outcome improvement may require additional intervention beyond feedback alone.

## Additional files

Additional file 1:
**The TIDieR (Template for Intervention Description and Replication) Checklist.** Information to include when describing an intervention and the location of the information. Additional file 2:
**Segmented regression on interrupted time series comparing organization 1 (mature use of RAI data) with organization 2 (recent adoption of RAI).** Additional analyses for interested readers.
