# Supplementary material for: Data for improvement and clinical excellence: report of an interrupted time series trial of feedback in long-term care
Source: Implement Sci. 2014 Nov 11;9:161. doi: 10.1186/s13012-014-0161-5 (PMC4230368; doi:10.1186/s13012-014-0161-5)
Supplement: Additional file 2: — Segmented regression on interrupted time series comparing organization 1 (mature use of RAI data) with organization 2 (recent adoption of RAI). Additional analyses for interested readers. [file 13012_2014_161_MOESM2_ESM.pdf]

Segmented Regression on Interrupted Time Series comparing Organization 1 (mature use of RAI data) with Organization 2 (recent adoption of RAI)

```
      log:  e:\segreg, 20120510.log
 log type:  text
opened on:  10 May 2012, 10:59:01
```

A. Both organizations combined

1) fall

```
. prais fall b1 b2 b3 b4 b5

Iteration 0:  rho = 0.0000
Iteration 1:  rho = -0.5861
Iteration 2:  rho = -0.6174
Iteration 3:  rho = -0.6176
Iteration 4:  rho = -0.6176
Iteration 5:  rho = -0.6176

Prais-Winsten AR(1) regression -- iterated estimates

      Source |       SS       df       MS              Number of obs =      25
-------------+------------------------------           F(  5,    19) =   27.17
       Model |  680.759987     5  136.151997           Prob > F      =  0.0000
    Residual |  95.1960183    19  5.01031675           R-squared     =  0.8773
-------------+------------------------------           Adj R-squared =  0.8450
       Total |  775.956005    24  32.3315002           Root MSE      =  2.2384

------------------------------------------------------------------------------
        fall |      Coef.   Std. Err.      t    P>|t|     [95% Conf. Interval]
-------------+----------------------------------------------------------------
          b1 |  -.5049905   .3940173    -1.28   0.215    -1.329678    .3196972
          b2 |   1.459094   1.492823     0.98   0.341    -1.665421    4.583609
          b3 |   .9264297   .4007524     2.31   0.032     .0876453    1.765214
          b4 |  -1.410525   1.736931    -0.81   0.427    -5.045965    2.224914
          b5 |  -.1566811   .4007524    -0.39   0.700    -.9954656    .6821034
       _cons |   20.89381   1.524642    13.70   0.000      17.7027    24.08492
-------------+----------------------------------------------------------------
         rho |  -.6175957
------------------------------------------------------------------------------
Durbin-Watson statistic (original)    3.165902
Durbin-Watson statistic (transformed) 2.659501
```

2) pain

```
. regress pain b1 b2 b3 b4 b5

      Source |       SS       df       MS              Number of obs =      25
-------------+------------------------------           F(  5,    19) =   10.52
       Model |  1263.78947     5  252.757894           Prob > F      =  0.0001
    Residual |  456.432276    19  24.0227514           R-squared     =  0.7347
-------------+------------------------------           Adj R-squared =  0.6648
       Total |  1720.22175    24  71.6759062           Root MSE      =  4.9013

------------------------------------------------------------------------------
        pain |      Coef.   Std. Err.      t    P>|t|     [95% Conf. Interval]
-------------+----------------------------------------------------------------
          b1 |   .1137145   1.171635     0.10   0.924    -2.338546    2.565975
          b2 |    -14.323   4.571533    -3.13   0.005    -23.89133   -4.754676
          b3 |  -.5419563   1.226671    -0.44   0.664    -3.109408    2.025496
          b4 |   3.801195   5.236351     0.73   0.477    -7.158615      14.761
          b5 |   .8090988   1.226671     0.66   0.517    -1.758353    3.376551
       _cons |   34.75533   4.562863     7.62   0.000     25.20515    44.30551
```

```
------------------------------------------------------------------------------

. estat dwatson

Durbin-Watson d-statistic(  6,    25) =  1.989333

. dwe

Durbin-Watson test with normal p value
----------------------------------------------------
       dw            Prob < dw          Prob > dw
----------------------------------------------------
     1.9893           0.1250             0.8750
----------------------------------------------------
```

3) <u>depression</u>

```
. regress depression b1 b2 b3 b4 b5

      Source |       SS       df       MS              Number of obs =      25
-------------+------------------------------           F(  5,    19) =   10.42
       Model |  792.685051     5   158.53701           Prob > F      =  0.0001
    Residual |  289.177078    19  15.2198462           R-squared     =  0.7327
-------------+------------------------------           Adj R-squared =  0.6624
       Total |  1081.86213    24  45.0775887           Root MSE      =  3.9013

------------------------------------------------------------------------------
  depression |      Coef.   Std. Err.      t    P>|t|     [95% Conf. Interval]
-------------+----------------------------------------------------------------
          b1 |  -1.860286      .93258    -1.99   0.061    -3.812198    .0916266
          b2 |  -4.443773    3.638778    -1.22   0.237    -12.05982    3.172278
          b3 |   3.387978    .9763867     3.47   0.003     1.344377    5.431579
          b4 |  -1.988513     4.16795    -0.48   0.639    -10.71213    6.735107
          b5 |  -1.418263    .9763867    -1.45   0.163    -3.461864    .6253375
       _cons |   42.92933    3.631877    11.82   0.000     35.32773    50.53094
------------------------------------------------------------------------------
. estat dwatson

Durbin-Watson d-statistic(  6,    25) =  2.104927

. dwe

Durbin-Watson test with normal p value
----------------------------------------------------
       dw            Prob < dw          Prob > dw
----------------------------------------------------
     2.1049           0.2000             0.8000
----------------------------------------------------
```

B. Organization 1 (more mature use of RAI data)

1) fall

```
. regress fall b1 b2 b3 b4 b5

      Source |       SS       df       MS              Number of obs =      25
-------------+------------------------------           F(  5,    19) =    1.60
       Model |  127.579525     5  25.5159049           Prob > F      =  0.2077
    Residual |  302.744758    19  15.9339346           R-squared     =  0.2965
-------------+------------------------------           Adj R-squared =  0.1113
       Total |  430.324283    24  17.9301784           Root MSE      =  3.9917

------------------------------------------------------------------------------
        fall |      Coef.   Std. Err.      t    P>|t|     [95% Conf. Interval]
-------------+----------------------------------------------------------------
          b1 |  -1.048571   .9542068    -1.10   0.286    -3.045749    .9486063
          b2 |   2.290404   3.723163     0.62   0.546    -5.502265    10.08307
          b3 |   1.344945   .9990294     1.35   0.194    -.7460475    3.435937
          b4 |  -1.780498   4.264606    -0.42   0.681    -10.70642    7.145425
          b5 |   .6256264   .9990294     0.63   0.539    -1.465366    2.716619
       _cons |   21.61333   3.716101     5.82   0.000     13.83544    29.39122
------------------------------------------------------------------------------
. estat dwatson

Durbin-Watson d-statistic(  6,    25) =  2.906879

. dwe

Durbin-Watson test with normal p value
----------------------------------------------------
       dw            Prob < dw          Prob > dw
----------------------------------------------------
     2.9069           0.9207             0.0793
----------------------------------------------------
```

2) pain

```
. regress pain b1 b2 b3 b4 b5

      Source |       SS       df       MS              Number of obs =      25
-------------+------------------------------           F(  5,    19) =    7.62
       Model |  1478.02725     5  295.605449           Prob > F      =  0.0004
    Residual |  737.219407    19  38.8010214           R-squared     =  0.6672
-------------+------------------------------           Adj R-squared =  0.5796
       Total |  2215.24665    24  92.3019439           Root MSE      =   6.229

------------------------------------------------------------------------------
        pain |      Coef.   Std. Err.      t    P>|t|     [95% Conf. Interval]
-------------+----------------------------------------------------------------
          b1 |   1.581714   1.489027     1.06   0.301    -1.534855    4.698284
          b2 |  -18.99339   5.809946    -3.27   0.004    -31.15374   -6.833033
          b3 |  -2.062868   1.558972    -1.32   0.201    -5.325834    1.200098
          b4 |    7.68877   6.654861     1.16   0.262    -6.240014    21.61755
          b5 |    .678868   1.558972     0.44   0.668    -2.584098    3.941834
       _cons |   38.84733   5.798927     6.70   0.000     26.71004    50.98463
------------------------------------------------------------------------------

. estat dwatson

Durbin-Watson d-statistic(  6,    25) =  2.299409

. dwe

Durbin-Watson test with normal p value
----------------------------------------------------
```

```
       dw            Prob < dw          Prob > dw
----------------------------------------------------
     2.2994           0.3743             0.6257
----------------------------------------------------
```

3) depression

```
. regress depression b1 b2 b3 b4 b5

      Source |       SS       df       MS              Number of obs =      25
-------------+------------------------------           F(  5,    19) =    3.52
       Model |  392.971405     5  78.5942809           Prob > F      =  0.0203
    Residual |  424.466226    19  22.3403277           R-squared     =  0.4807
-------------+------------------------------           Adj R-squared =  0.3441
       Total |  817.437631    24  34.0599013           Root MSE      =  4.7266

------------------------------------------------------------------------------
  depression |      Coef.   Std. Err.      t    P>|t|     [95% Conf. Interval]
-------------+----------------------------------------------------------------
          b1 |   .1277145   1.129863     0.11   0.911    -2.237116    2.492545
          b2 |  -8.521593   4.408545    -1.93   0.068    -17.74878     .705597
          b3 |   .9817909   1.182937     0.83   0.417    -1.494124    3.457706
          b4 |   .2737377    5.04966     0.05   0.957    -10.29532     10.8428
          b5 |  -1.320934   1.182937    -1.12   0.278    -3.796849    1.154981
       _cons |     46.888   4.400184    10.66   0.000     37.67831    56.09769
------------------------------------------------------------------------------

. estat dwatson

Durbin-Watson d-statistic(  6,    25) =  2.520066



. dwe

Durbin-Watson test with normal p value
----------------------------------------------------
       dw            Prob < dw          Prob > dw
----------------------------------------------------
     2.5201           0.6109             0.3891
----------------------------------------------------
```

C. Organization 2 (recent adoption of RAI assessments)

1) fall

```
. regress fall b1 b2 b3 b4 b5

      Source |       SS       df       MS              Number of obs =      25
-------------+------------------------------           F(  5,    19) =    0.81
       Model |  157.518316     5  31.5036631           Prob > F      =  0.5562
    Residual |  737.968174    19  38.8404302           R-squared     =  0.1759
-------------+------------------------------           Adj R-squared = -0.0410
       Total |   895.48649    24  37.3119371           Root MSE      =  6.2322

------------------------------------------------------------------------------
        fall |      Coef.   Std. Err.      t    P>|t|     [95% Conf. Interval]
-------------+----------------------------------------------------------------
          b1 |  -.7611428   1.489783    -0.51   0.615    -3.879294    2.357009
          b2 |   5.167088   5.812895     0.89   0.385    -6.999442    17.33362
          b3 |   1.080538   1.559763     0.69   0.497    -2.184084    4.345161
          b4 |   -1.98504    6.65824    -0.30   0.769     -15.9209    11.95082
          b5 |  -.5345384   1.559763    -0.34   0.736    -3.799161    2.730084
       _cons |     23.564   5.801871     4.06   0.001     11.42054    35.70746
------------------------------------------------------------------------------
. estat dwatson

Durbin-Watson d-statistic(  6,    25) =  2.725266

. dwe

Durbin-Watson test with normal p value
----------------------------------------------------
       dw            Prob < dw          Prob > dw
----------------------------------------------------
     2.7253           0.8061             0.1939
----------------------------------------------------
```

2) pain

```
. regress pain b1 b2 b3 b4 b5

      Source |       SS       df       MS              Number of obs =      25
-------------+------------------------------           F(  5,    19) =   13.55
       Model |  937.112819     5  187.422564           Prob > F      =  0.0000
    Residual |  262.757409    19  13.8293373           R-squared     =  0.7810
-------------+------------------------------           Adj R-squared =  0.7234
       Total |  1199.87023    24  49.9945928           Root MSE      =  3.7188

------------------------------------------------------------------------------
        pain |      Coef.   Std. Err.      t    P>|t|     [95% Conf. Interval]
-------------+----------------------------------------------------------------
          b1 |      -.042   .8889589    -0.05   0.963    -1.902612    1.818612
          b2 |  -13.95372   3.468576    -4.02   0.001    -21.21353   -6.693906
          b3 |  -.0094835   .9307165    -0.01   0.992    -1.957496    1.938529
          b4 |  -.2629962   3.972996    -0.07   0.948    -8.578572    8.052579
          b5 |   .3046263   .9307165     0.33   0.747    -1.643386    2.252638
       _cons |   22.45533   3.461997     6.49   0.000     15.20929    29.70138
------------------------------------------------------------------------------

. estat dwatson

Durbin-Watson d-statistic(  6,    25) =  1.997092

. dwe

Durbin-Watson test with normal p value
----------------------------------------------------
```

```
       dw            Prob < dw          Prob > dw
----------------------------------------------------
     1.9971           0.1293             0.8707
----------------------------------------------------
```

3) depression

```
. regress depression b1 b2 b3 b4 b5

      Source |       SS       df       MS              Number of obs =      25
-------------+------------------------------           F(  5,    19) =   11.03
       Model |  1861.67762     5  372.335524           Prob > F      =  0.0000
    Residual |  641.203844    19  33.7475708           R-squared     =  0.7438
-------------+------------------------------           Adj R-squared =  0.6764
       Total |  2502.88146    24  104.286728           Root MSE      =  5.8093

------------------------------------------------------------------------------
  depression |      Coef.   Std. Err.      t    P>|t|     [95% Conf. Interval]
-------------+----------------------------------------------------------------
          b1 |  -2.323143    1.38868    -1.67   0.111    -5.229684    .5833983
          b2 |  -6.255348   5.418408    -1.15   0.263    -17.59621    5.085511
          b3 |   4.714571   1.453912     3.24   0.004     1.671499    7.757643
          b4 |  -5.319033   6.206384    -0.86   0.402    -18.30914    7.671079
          b5 |  -2.023715   1.453912    -1.39   0.180    -5.066786    1.019357
       _cons |   29.96267   5.408132     5.54   0.000     18.64332    41.28202
------------------------------------------------------------------------------

. estat dwatson

Durbin-Watson d-statistic(  6,    25) =  2.380314



. dwe

Durbin-Watson test with normal p value
----------------------------------------------------
       dw            Prob < dw          Prob > dw
----------------------------------------------------
     2.3803           0.4595             0.5405
----------------------------------------------------
```
